# Monitoring Ammonium Polyphosphate (APP) Biodegradation by *Acinetobacter nosocomialis* D-3 Using DAPI

**DOI:** 10.3390/molecules29112667

**Published:** 2024-06-05

**Authors:** Xiangxiang Li, Yule Cai, Qiqing Qiu, Jiamin Wu, Jing Wang, Jieqiong Qiu

**Affiliations:** 1 College of Life Sciences and Medicine, Zhejiang Sci-Tech University, Hangzhou 310018, China; 2 Hangzhou JLS Flame Retardants Chemical Co., Ltd., Hangzhou 310011, China

**Keywords:** APP, DAPI dye, fluorescence, flame retardants, microbial degradation

## Abstract

Ammonium polyphosphate (APP), a pivotal constituent within environmentally friendly flame retardants, exhibits notable decomposition susceptibility and potentially engenders ecological peril. Consequently, monitoring the APP concentration to ensure product integrity and facilitate the efficacious management of wastewater from production processes is of great significance. A fluorescent assay was devised to swiftly discern APP utilizing 4′,6′-diamino-2-phenylindole (DAPI). With increasing APP concentrations, DAPI undergoes intercalation within its structure, emitting pronounced fluorescence. Notably, the flame retardant JLS-PNA220-A, predominantly comprising APP, was employed as the test substrate. Establishing a linear relationship between fluorescence intensity (F-F0) and JLS-PNA220-A concentration yielded the equation y = 76.08x + 463.2 (R^2^ = 0.9992), with a LOD determined to be 0.853 mg/L. The method was used to assess the degradation capacity of APP-degrading bacteria. Strain D-3 was isolated, and subsequent analysis of its 16S DNA sequence classified it as belonging to the *Acinetobacter* genus. *Acinetobacter nosocomialis* D-3 demonstrated superior APP degradation capabilities under pH 7 at 37 °C, with degradation rates exceeding 85% over a four-day cultivation period. It underscores the sensitivity and efficacy of the proposed method for APP detection. Furthermore, *Acinetobacter nosocomialis* D-3 exhibits promising potential for remediation of residual APP through environmental biodegradation processes.

## 1. Introduction

Flame retardants, including halogenated flame retardants (HFRs) and halogen-free flame retardants (HFFRs), have been extensively utilized to mitigate the occurrence of fires and minimize property damage [1]. While HFRs present significant environmental and health hazards due to their release of toxic smoke and gaseous hydrogen halides during combustion, HFFRs have emerged as rapid alternatives, characterized by their efficiency, non-toxicity, and environmental compatibility [2,3]. Ammonium polyphosphate (APP), a pivotal component of HFFRs, has garnered substantial attention for its notable biological safety profile and cost-effectiveness [4]. Within many formulations of APP-based flame retardants, APP assumes a primary role, serving as both an acid and gas source, thereby determining the formulation’s fire prevention efficacy based on APP quality and abundance [5]. However, APP is prone to degradation, reducing its effectiveness as a flame retardant [6]. Additionally, APP exhibits biological toxicity to aquatic organisms like algae and crustaceans. The discharge of APP also causes eutrophication of water sources, which leads to severe water blooms and poses serious hazards to the survival of aquatic organisms [7,8]. Therefore, it is imperative to devise a rapid and straightforward method for detecting APP concentration to monitor its wastewater presence and ultimately decrease the APP concentration.

APP, a polyphosphate (PolyP) variant, is composed of PolyP and ammonium ions and has prompted the exploration of diverse detection methodologies. Techniques such as ^31^P nuclear magnetic resonance spectroscopy (^31^P-NMR) [9,10], ion chromatography (IC) [11], and capillary gel electrophoresis [12,13] have demonstrated commendable sensitivity and precision in PolyP detection. However, their application is encumbered by intricate sample preconditioning procedures and laborious, time-intensive detection processes requiring bulky instrumentation. Thus, the imperative arises to develop a novel detection system capable of achieving rapid, accurate, and cost-effective assessment of APP concentrations in practical production settings. Fluorescence detection technology has emerged as a promising avenue, lauded for its heightened sensitivity, specificity, low detection costs, and user-friendly operation [14]. DAPI, as an intercalated dye, has emerged as a cornerstone in DNA detection, especially for AT-rich sequences, manifesting at 450 nm when the excitation light is at 360 nm [15,16]. Moreover, DAPI has been leveraged in PolyP detection to monitor PolyP metabolism within cells to form the DAPI/PolyP complexes [17]. The complex modulates the fluorescence signal of DAPI, culminating in a characteristic emission peak at 550 nm [18,19]. Given the specific recognition between DAPI and PolyPs, the robust fluorescence alteration of DAPI is a promising avenue for APP detection.

To mitigate the environmental ramifications associated with APP, various adsorbents, such as bentonite and biochar, have been employed for phosphate removal. However, challenges persist in the degradation and recycling of phosphate resources [20,21,22]. Chemical phosphorus removers, including aluminum salts, iron salts, and lime, demonstrate high efficacy in degradation capability. Nonetheless, these chemicals often pose environmental risks, leading to secondary pollution of ecological systems [23,24]. The discovery of phosphate-accumulating organisms (PAOs) has introduced a biodegradation approach utilizing microbial phosphorus removal, holding promising prospects for widespread application [25,26]. Numerous genera of bacteria, such as *Acinetobacter*, *Aeromonas*, and *Corynebacterium*, belong to PAOs. Studies have demonstrated that PAOs can produce and secrete many organic acids (e.g., gluconic acid, pyruvate acid, citric acid) and phosphatases to facilitate the degradation of polyphosphate [27]. The resulting degradation products serve as developmental and energy sources, while the accumulated phosphates within PAOs are removed through the disposal of excess sludge at the treatment’s culmination [28]. Hence, biodegradation emerges as a cost-effective and environmentally benign industrial wastewater treatment technology, offering potential solutions to mitigate the ecological and environmental challenges stemming from APP in flame-retardant industry wastewater.

This study first presents a swift and convenient detection system founded upon the DAPI/APP complex for quantifying APP concentration to restore wastewater in flame-retardant production effluents. Sensitivity assessments revealed a limit of detection (LOD) for APP detection at 3.079 μg/L. To evaluate the system’s applicability in assessing the integrity of APP-based flame retardants, namely JLS-PNA220-A, predominantly comprising around 95% mixed PolyP salts, with 20–30% APP (n > 1000) as the main PolyP salt. A linear correlation was observed between JLS-PNA220-A concentration and DAPI fluorescence intensity. The assay was then applied to isolate efficient APP-degrading strains (*Acinetobacter nosocomialis* D-3) from activated sewage sludge samples from JLS Chemical Flame Retardant Co., Ltd. and monitor APP levels in the culture environment. Based on the fluorescence detection system, a biodegradation approach was constructed to address environmental contamination stemming from APP. *Acinetobacter nosocomialis* D-3 was tested to ascertain its efficacy in degrading APP under varied temperatures, pH, and phosphorus sources.

## 2. Results and Discussion

### 2.1. Analysis of APP Concentration by DAPI

The evaluation of optimal incubation parameters encompassed the meticulous observation of DAPI fluorescence intensity at 550 nm over various reaction durations. As depicted in Figure S1, a continuous escalation in fluorescence intensity was noted as the reaction time progressed from 1 to 5 min, culminating in the attainment of maximal fluorescence intensity at the 5 min mark. To strike a harmonious balance between the imperatives of expeditiousness and precision inherent in industrial wastewater analysis, it was deduced that 5 min serves as the optimal reaction duration for DAPI within the APP detection. The timeframe ensures the optimal augmentation of fluorescence intensity while preserving the swift detection pace. Subsequent experiments were conducted adhering to an incubation period of 5 min in the context of APP detection.

To investigate the sensitivity of the detection of varying concentrations of APP, a series of meticulously prepared APP solutions were subjected to gradient dilution for subsequent fluorescence analysis. As illustrated in Figure S2a, in the absence of APP, DAPI exhibits its inherent emission fluorescence peak at 475 nm. However, with the gradual increase in APP concentrations, the emission fluorescence intensities at 550 nm become markedly more pronounced than the peak at 475 nm. It delineates the emergence of a distinct peak characteristic of the DAPI/PolyP complex formation. The *N*-containing indole ring of DAPI intricately interacts with the O atoms of polyphosphate, resulting in the emission fluorescence change in DAPI at 550 nm [18]. Subsequently, the declining intensity at 475 nm signifies a complete reaction between residual monomeric DAPI and APP, culminating in maximal fluorescence intensity at 550 nm.

The fluorescence intensity at 550 nm of DAPI exhibits a gradual increase corresponding to the escalating concentration of APP, as illustrated in Figure S2b. When APP concentrations span from 3.079 to 300 μg/L, a linear correlation emerges between the concentration of APP and the intensity of DAPI fluorescence. The relationship is encapsulated by the equation y = 4.576x + 302.6, where y represents relative fluorescence intensity (F-F0) and x signifies the concentration of APP in the solution. The commendable coefficient of determination (R^2^ = 0.9906) attests to the system’s adeptness in discerning and quantifying APP concentrations. Employing the 3σ/slope analysis (σ = 4.697 μg/L, slope = 4.576), the LOD for APP solution is determined to be 3.079 μg/L. Thus, it offers a practical and productive means for monitoring the concentration of APP in environmental and industrial samples. In addition, owing to the degradability of APP, the detection system can also serve as a tool for the quality assessment of several APP products, such as APP-based flame retardants.

### 2.2. Analysis of APP in JLS-PNA220-A by DAPI

**Figure 1:**
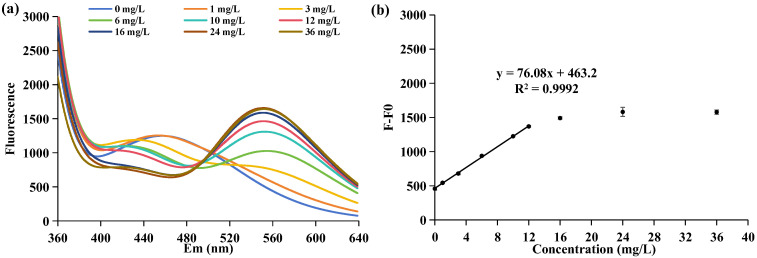
The fluorescence emission intensity of DAPI with concentrations of JLS-PNA220-A ranged from 0 to 36 mg/L. The concentrations of JLS-PNA220-A were as follows: 0, 1, 3, 6, 10, 12, 16, 24, and 36 mg/L. (**a**) The fluorescence emission spectra of DAPI range from 360 to 640 nm. (**b**) The linear relationship between the relative fluorescence intensity (FF0) at 550 nm and JLS-PNA220-A concentration (0–12 mg/L). The final concentration of DAPI is 0.5 μM. *Ex* = 330 nm; *Em* = 550 nm.

To assess the method’s feasibility in detecting APP-based flame-retardant qualities, we investigated the correlation between fluorescence intensity and APP concentration of the APP-based flame retardant JLS-PNA220-A. Considering the microbial requirement for APP degradation within JLS-PNA220-A, the JLS-PNA220-A sample was dissolved in an R2A liquid medium. As anticipated, the APP-based flame retardant JLS-PNA220-A elicited alterations in DAPI fluorescence in Figure 1a, mirroring similar fluorescence emission spectra at 550 nm as observed for the APP/PolyP complex in Figure S2a. In the absence of JLS-PNA220-A, there was a characteristic peak at 475 nm caused by the DAPI fluorescence signal, consistently exhibiting a blue shift and decay with increasing JLS-PNA220-A concentration. Upon reaching an excess of JLS-PNA220-A, a robust fluorescence signal at 550 nm emerged, reaching maximal intensity as the JLS-PNA220-A concentration reached 12 mg/L. It was simultaneously accompanied by a fluorescence signal at 420 nm due to the R2A medium. It suggests that the APP of JLS-PNA220-A can bind with DAPI to form DAPI/PolyP structures, offering a pathway for assessing APP quality in JLS-PNA220-A.

Regarding APP detection in JLS-PNA220-A, the maximal emission intensity at 550 nm exhibited a proportional increase with the elevation in JLS-PNA220-A concentration, as depicted in Figure 1b. Across the JLS-PNA220-A concentration range of 0–12 mg/L, the change in fluorescence intensity (F-F0) displayed a linear relationship with the JLS-PNA220-A concentration. The concentration of JLS-PNA220-A can be ascertained using the equation y = 76.08x + 463.2, where y represents fluorescence intensity and x represents JLS-PNA220-A concentration. The coefficient of determination (R^2^) calculated for this equation was 0.9992, indicating a high correlation between the relative fluorescence intensity (F-F0) and JLS-PNA220-A concentration. Moreover, it exhibited a LOD of 0.856 mg/L based on the 3σ/slope analysis; σ is 21.71 mg/L, and the slope is 76.08.

Since flame retardants constitute a uniform mixture, the proportion of APP within the flame retardant remains consistent, establishing a direct correlation between APP concentration and flame retardant concentration. The concentration of the flame retardant can be accurately calculated by utilizing the linear relationship derived from the interaction between APP and DAPI, coupled with the fluorescence intensity variation. The approach enables the assessment of the efficiency and integrity of APP flame-retardant products. Significantly, leveraging fluorescence detection technology, the fluorescent agent DAPI forms linear relationships with the concentrations of APP-based JLS-PNA220-A, demonstrating remarkable detection sensitivity. Consequently, it holds promise for environmental or industrial APP detection in wastewater. Therefore, this method was used as an efficient APP concentration monitoring method for selecting APP-dominant degrading bacteria to detect and remediate polluted water sources.

### 2.3. Isolation and Identification of the APP-Degradation Strain

**Figure 2:**
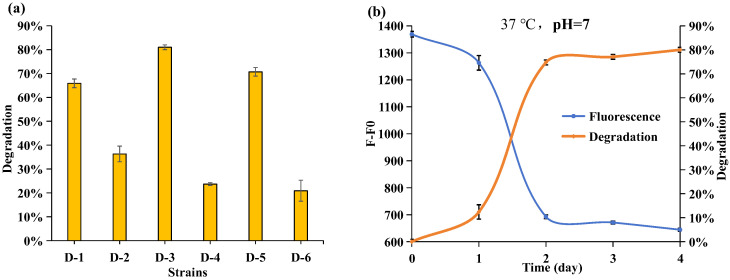
(**a**) APP degradation ability of different isolated strains, from strain D-1 to strain D-6. (**b**) The variation in fluorescence intensity (F-F0) at 550 nm and APP degradation as the strain D-3 was incubated for 4 days. The initial concentration of JLS-PNA220-A was 12 mg/L, and the final concentration of DAPI was 0.5 μM. *Ex* = 330 nm, *Em* = 550 nm. The blue line was the fluorescence intensity. The orange line was the degradation rate.

To effectively address the management of wastewater generated in APP-based flame retardant production and the residual presence of APP in the environment, microbial degradation emerges as a promising environmental remediation method. In isolating efficient bacteria, six colonies (D-1~D-6) were isolated and purified from R2A culture media. Subsequently, their abilities to degrade APP in JLS-PNA220-A were studied individually, leveraging the equation y = 76.08x + 463.2 developed in Figure 2b to calculate the APP concentration in JLS-PNA220-A and APP degradation rate (Degradation rate = (C_(JLS-PNA220-A initial)_ − C_(JLS-PNA220-A test)_)/C_(JLS-PNA220-A initial)_). During the 24-h monitoring assays, strain D-3 exhibited the highest degradation capacity, as illustrated in Figure 2a. Within the initial two days of incubation, the fluorescence intensity of DAPI decreased rapidly, with over 75% of the APP in the medium being degraded by strain D-3 (Figure 2b). It suggests rapid growth in the bacterial population, efficiently utilizing a significant quantity of APP as a phosphate resource for growth and development. In the subsequent two days of incubation, the degradation rates remained stable, indicating a sustained level of APP concentration and slower bacterial propagation. Overall, strain D-3 demonstrated an impressive capacity for APP degradation within a short-term incubation period, highlighting its significance for achieving high-efficiency eco-recovery.

**Figure 3:**
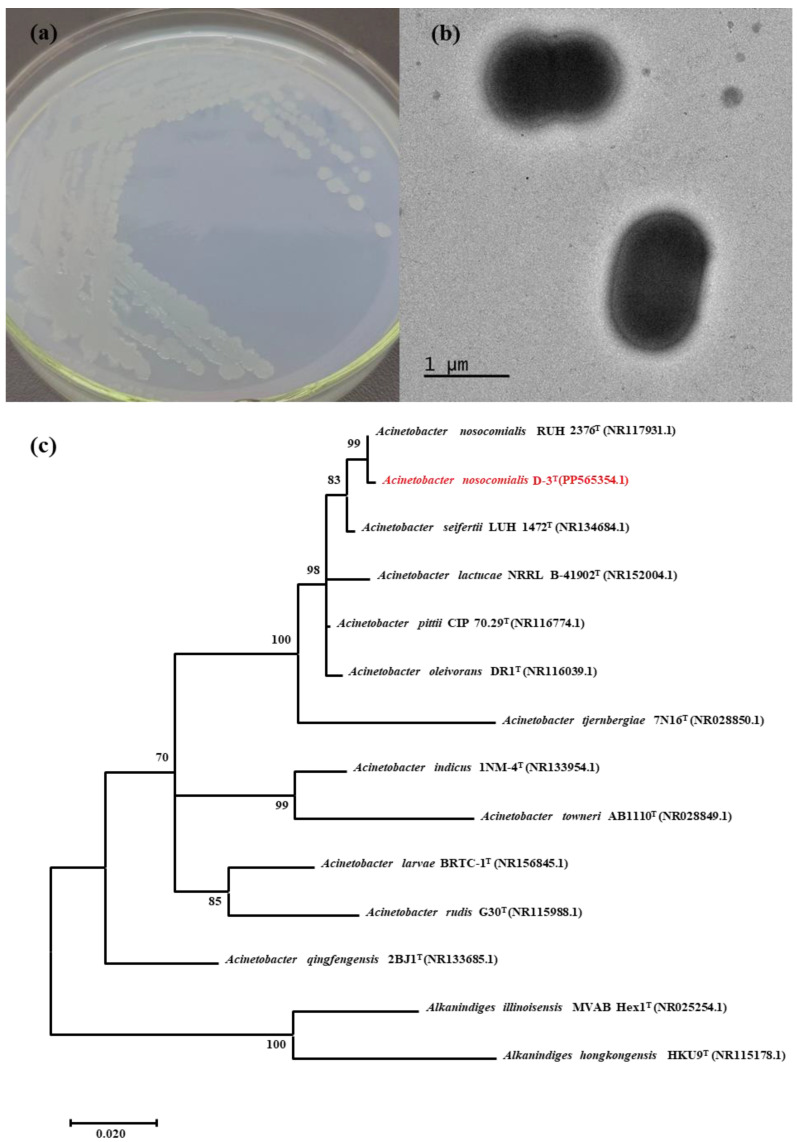
Morphological structure of the strain D-3 in R2A agar medium: (**a**) Characters of colonies after one-day culture; (**b**) Characteristics of strain D-3 cells through the TEM; the magnification is ×10,000; (**c**) Neighbor-joining tree of *Acinetobacter nosocomialis* D-3 developed based on the 16S rDNA sequence in MEGA 11.

Certain microorganisms, known as phosphate-accumulating organisms (PAOs), can degrade polyphosphate and accumulate phosphorus for growth. These microorganisms include *Accumulibacter*, *Acinetobacter*, *Dechloromonas*, and *Zoogloea*. As depicted in Figure 3a, strain D-3 was streaked on R2A agar medium for one day in a 37 °C incubator, forming round, moist, and white colonies. The Gram stain exhibited in Figure S3 indicates that strain D-3 is a Gram-negative strain. The cell morphologies of strain D-3 were observed using TEM, as illustrated in Figure 3b. It revealed characteristic features of a ball-rod-shaped morphology with a capsule, without flagellum, and arranged singly or in pairs. Upon comparing these morphology characteristics, it was found that the traits of strain D-3 align with the typical features of the *Acinetobacter* genus [29,30].

The analysis compared the 16S rDNA sequence of strain D-3 with DNA sequences using BLAST. The results revealed the highest homology between strain D-3 and *Acinetobacter nosocomialis*. Genetic sequences of similar strains were then selected and downloaded to construct a phylogenetic tree using the neighbor-joining method, as illustrated in Figure 3c. The tree demonstrated a clear linkage relationship, placing the isolated strain D-3 within the genus *Acinetobacter*. Based on these findings, it can be concluded that strain D-3 belongs to the *Acinetobacter nosocomialis* species. The 16S rDNA sequence of strain D-3 has been deposited in the NCBI database with the GenBank accession number PP565354. *Acinetobacter*, as part of the PAOs group, exhibits a robust metabolism of polyphosphates. In aerobic conditions, *Acinetobacter* absorbs phosphorus from the environment and stores it as PolyP for energy storage. PolyPs are hydrolyzed to ATP without oxygen, serving as an energy source [31,32]. Since the 1980s, *Acinetobacter* has been recognized as a highly efficient bacterium for phosphorus removal and has played a significant role in wastewater treatment processes [33]. *Acinetobacter*’s release of organic acids and phosphatases facilitates the breakdown of PolyP, releasing soluble phosphate [34,35]. Thus, *Acinetobacter nosocomialis* D-3 holds promise as an efficient bacterium for degrading APP and can be utilized to remedy APP-based flame retardants.

### 2.4. Effect of the Phosphorus Resource

**Figure 4:**
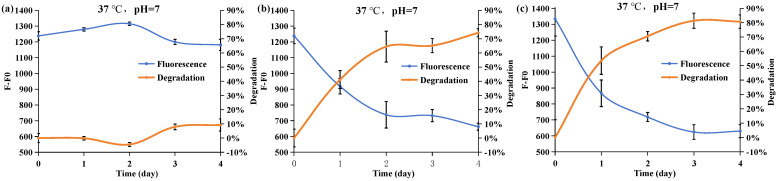
(**a**) The fluorescence intensity (F-F0) change in the R2A medium in the absence of K_2_HPO_4_ and *Acinetobacter nosocomialis* D-3 during the four-day culture; the effect of phosphorus resource on DAPI fluorescence intensity (F-F0) and degradation rate when (**b**) K_2_HPO_4_ is absent and (**c**) K_2_HPO_4_ is present. The blue line is the fluorescence intensity. The orange line is the degradation rate of APP calculated based on fluorescence intensity.

K_2_HPO_4_ is commonly employed as a phosphorus source in bacterial culture media. To investigate the phosphate resources utilized by *Acinetobacter nosocomialis* D-3, an incubation process was conducted in the absence of K_2_HPO_4_. This allowed for the evaluation of *Acinetobacter nosocomialis* D-3’s ability to use alternative phosphate sources. In Figure 4a, when the R2A medium lacked both *Acinetobacter nosocomialis* D-3 and K_2_HPO_4_, the degradation rates of APP over four days were approximately 10%, suggesting that APP cannot be substantially hydrolyzed under short-term incubation without efficient APP degradation strain D-3. Moreover, the initial average fluorescence intensity indicates that the absence of K_2_HPO_4_ does not affect R2A fluorescence intensity. In Figure 4b,c, both groups of *Acinetobacter nosocomialis* D-3 exhibited a high ability to degrade APP. However, in the absence of K_2_HPO_4_, phosphate acquisition was limited, affecting the degradation system of *Acinetobacter nosocomialis* D-3. After four days of incubation, the APP degradation rates in the absence and presence of K_2_HPO_4_ were 74% and 81%, respectively. It indicates that when a directly accessible phosphorus source is absent from the environment, *Acinetobacter nosocomialis* D-3 decomposes APP for its life activities as a phosphorus source, limiting its growth.

### 2.5. Effect of Culture Temperature on the Degrading Ability of Acinetobacter nosocomialis D-3

**Figure 5:**
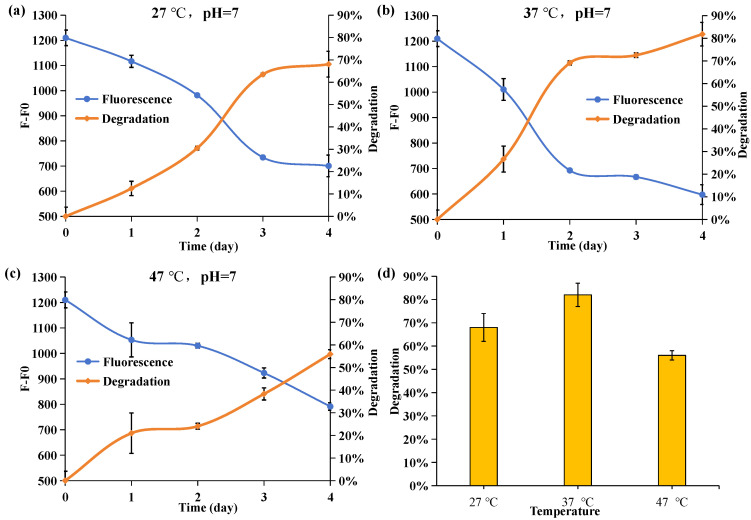
Effect of temperature on DAPI fluorescence intensity (F-F0) at 550 nm and APP degradation rate when the environment temperature was (**a**) 27 °C, (**b**) 37 °C, and (**c**) 47 °C. The blue line is the fluorescence intensity. The orange line is the degradation rate of APP calculated based on fluorescence intensity. (**d**) The degradation rate of *Acinetobacter nosocomialis* D-3 when cultured at 27 °C, 37 °C, and 47 °C on the fourth day.

The incubation temperatures for the three groups were set at 27 °C, 37 °C, and 47 °C, respectively. Figure 5 illustrates a divergence in the degradation trend and final concentration after four days of culture, highlighting the significant impact of temperature on the characteristics of *Acinetobacter nosocomialis* D-3. During the initial two days, APP degradation in the culture medium amounted to 31%, 69%, and 24%, respectively, with increasing culture temperature, indicating a notable discrepancy in the APP degradation capability. Figure 5d depicts the degradation of APP on the final day of culture. Notably, *Acinetobacter nosocomialis* D-3 incubated at 37 °C exhibited the highest APP degradation ability, approximately 82%. It underscores 37 °C as the optimal incubation temperature. Deviation from the optimal temperature restricts the cellular activities of *Acinetobacter nosocomialis* D-3 and affects the activities of phosphatases within *Acinetobacter nosocomialis* D-3. Collectively, these factors contribute to a decrease in the rate of APP degradation as the temperature strays from the optimum.

### 2.6. Effect of Initial pH Values on the Degrading Ability of Acinetobacter nosocomialis D-3

**Figure 6:**
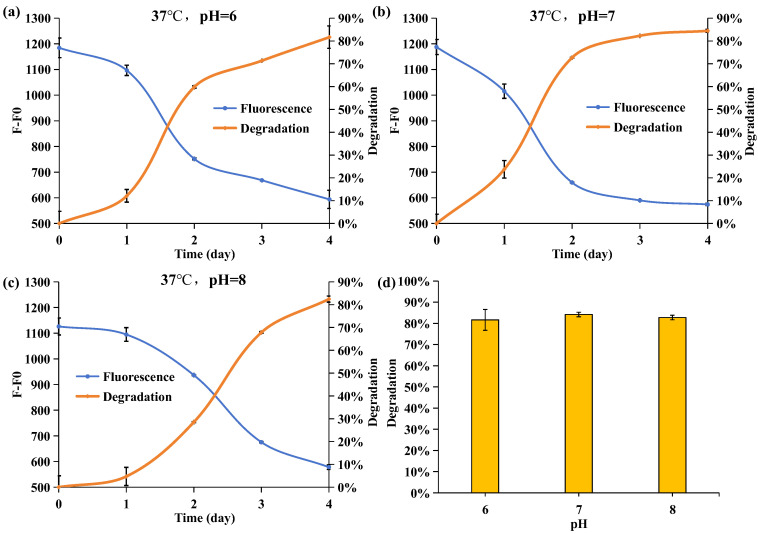
Effect of pH on DAPI fluorescence intensity (F-F0) at 550 nm and APP degradation rate when (**a**) pH = 6, (**b**) pH = 7, and (**c**) pH = 8. The blue line is the fluorescence. The orange line is the degradation rate. (**d**) The degradation rate of pH = 6, 7, 8 on the fourth day.

pH represents another pivotal factor influencing microbial behavior. As depicted in Figure 6, varying pH levels notably impact the APP degradation rate. Across pH ranges of 6 to 8, the degradation rates of APP after four days of incubation reached 81%, 85%, and 82%, respectively. However, compared to the groups with pH levels of 6 and 7, *Acinetobacter nosocomialis* D-3 required more time to degrade an equivalent amount of APP when the incubation pH was 8. Given that the secretion of organic acids, such as gluconic acid [34], is one of the fundamental mechanisms by which *Acinetobacter* decomposes PolyP, fluctuations in incubation pH significantly influence the efficiency of APP degradation. In an alkaline environment, organic acids are consumed in neutralizing reactions, resulting in a lower rate of APP degradation. Conversely, as acidity increases, the hydrolysis of APP intensifies. Coordinated with organic acids, it amplifies APP degradation. Moreover, a neutral environment is more conducive to *Acinetobacter* growth [36]. Thus, the results suggest that the pH value of the incubating environment equal to 7 is favorable for *Acinetobacter nosocomialis* D-3 in degrading APP.

To accurately obtain the optimal culture conditions for *Acinetobacter nosocomialis* D-3 to degrade APP, orthogonal experiments were conducted on the two conditions of temperature and pH. From Table S1, the effect of temperature on the degradation rate of *Acinetobacter nosocomialis* D-3 was more significant. At four days of incubation, the 37 °C group generally exhibited the highest degradation efficiency. Comparing the effects of pH, the best degradation effect was shown in group pH 7. In the experiment, the highest APP degradation effect of *Acinetobacter nosocomialis* D-3 was obtained at pH 7 and 37 °C.

## 3. Materials and Methods

### 3.1. Chemicals

The following chemicals and their respective supply companies were utilized in this study: 4′,6′-diamino-2-phenylindole (DAPI) (5 mg/mL) (Beyotime Biotechnology Co., Ltd., Shanghai, China), ammonium polyphosphate (n > 1000) (Acmec Biochemical Co., Ltd., Shanghai, China), JLS-PNA220-A flame retardant (JLS Chemical Flame Retardant Co., Ltd., Hangzhou, China), and biowest agarose (Baygene Biotechnologies Co., Ltd., Shanghai, China). The R2A liquid medium comprised D-(+)-Glucose (USP grade), starch soluble (BC grade), magnesium sulfate anhydrous (>98%), sodium pyruvate (>99%), potassium phosphate dibasic anhydrous (>98%), tryptone (FMB grade), casein peptone type II (FMB grade), yeast extract (FMB grade), and peptone (FMB grade) (Sangon Biotech Co., Ltd., Shanghai, China). Smart Plus E ultrapure water meter (Heal Force Bio-meditech Co., Ltd., Shanghai, China)-filtered ultra-clean water was utilized throughout the experiment.

### 3.2. Apparatus

The solid media and liquid media were respectively incubated in the water-jacket constant temperature incubator GSP-9270MBE (Boxun Co., Ltd., Shanghai, China) and the shaker incubator TS-200B (Tiancheng Experimental Instrument Manufacturing Co., Ltd., Shanghai, China). The morphological characteristics of the isolated strain were observed under a transmission electron microscope (TEM). The fluorescence intensity was assessed using the fluorescence spectrometer F-4600 (Hitachi, Tokyo, Japan). Measurements were conducted under reaction conditions maintained at 30 °C in darkness. Emission spectral data were recorded within the 360–640 nm range, with an excitation wavelength of 330 nm. The maximum emission (Em_max_) peak was observed at 550 nm, where fluorescence intensities were recorded. Both excitation and emission slit widths were set to 5 nm. The TMP voltage was maintained at 600 V for optimal performance.

### 3.3. Analysis of APP

To evaluate the optimal reaction time, 1 mL of 3 mg/L APP and 5 μL of 100 μM DAPI were combined in 1.5 mL centrifuge tubes. The reaction mixtures were thoroughly mixed and then incubated at 30 °C in darkness. Incubation times ranging from 1, 2, 3, 4, 5, 6, 7, and 8 min were examined. To assess APP detection sensitivity, 1 mL of various mixtures containing different concentrations of APP and 5 μL of 100 μM DAPI were prepared. The final APP concentrations ranged from 0, 30, 90, 150, 240, 300, 900, 1500, 2400, and 3000 μg/L, while the final DAPI concentration was maintained at 0.5 μM. The mixtures were vigorously shaken and then incubated in darkness for 5 min. Fluorescence intensity at 550 nm was detected by the fluorescence spectrometer F-4600 under a 330 nm excitation wavelength. Each sample underwent analysis at least three times.

### 3.4. JLS-PNA220-A Detection

Considering the biodegradation potential of JLS-PNA220-A, it was solubilized in the R2A liquid medium. A total of 3 mg of JLS-PNA220-A flame retardant was introduced into 250 mL of R2A liquid medium, where it underwent complete dissolution through agitation in a shaker at 30 °C and 220 rpm, resulting in a final concentration of 12 mg/L. Various JLS-PNA220-A solutions were prepared, spanning concentrations of 0, 1, 3, 6, 10, 12, 16, 24, and 36 mg/L. Subsequently, 1 mL of the JLS-PNA220-A solution was combined with 5 μL of 100 μM DAPI and mixed. The mixture was incubated for 5 min in darkness, followed by fluorescence analysis. Fluorescence intensity at 550 nm was detected by the fluorescence spectrometer F-4600 under a 330 nm excitation wavelength. Each sample underwent analysis at least three times.

### 3.5. Isolation, Enrichment, and Identification of Acinetobacter nosocomialis D-3

Activated sewage sludge samples, intended for isolating specific degrading APP strains, were procured from the sewage pool of JLS Chemical Flame Retardant Co., Ltd. These samples were solubilized in ddH_2_O in a 1:1 ratio by vigorous shaking and precipitated for 30 min. Subsequently, 0.5 mL of the supernatant gradient was subjected to dilution (10^−1^–10^−4^) and evenly spread onto R2A solid media, which were then incubated at 37 °C for 2 days. Following the incubation period, media exhibiting moderate colony density were selected, and individual colonies (D-1~D-6) were transferred to a sterile R2A solid medium to purify single colonies at 37 °C. This purification process was iterated at least three times to ensure the isolation of pure strains. The purified strains were systematically labeled and expanded in R2A liquid media.

To detect the APP degradation ability of isolated strains, a pre-sterilized R2A medium was employed to dissolve JLS-PNA220-A. The mixture was achieved by shaking at 220 rpm and 30 °C. Subsequently, the mixture was filtered using filters (0.22 μm). A total of 1 mL of the expanded strain solutions was added to the 30 mL sterilized R2A liquid media that contained 12 mg/L JLS-PNA220-A, and the mixtures were incubated at 37 °C and 220 rpm for 4 days. Every 24 h, 1 mL of the mixture was sampled and then centrifuged in a Centrifuge MiniSpin^®^ plus (Eppendorf, Hamburg, Germany) at 8000 rpm for 90 s. A total of 1 mL of samples were stored in a 4 °C refrigerator until the fourth day. Based on the detection system developed in Section 3.4, a uniform fluorescence assay was performed on the last day to calculate the remaining concentration of JLS-PNA220-A for stored samples. Based on this, the degradation rate of APP over four days can be reckoned, targeting high-APP degradation efficiency strains. Each sample was measured three times.

Strain D-3 was isolated with an efficient degradation capacity, streaked on R2A agar plates, and incubated at 37 °C for 1 day to observe the morphological characteristics of terminal single colonies. TEM was used to characterize the morphological attributes of strain D-3 cells; the magnification was ×10,000. For species identification, the purified colonies were sent to Sangon Biotech Co., Ltd. (Shanghai, China) and amplified 16S rDNA sequence by bacterial primers 27F and 1492R for sequencing. The alignment of the result was compared with other 16S rDNA sequences in the GenBank database (https://www.ncbi.nlm.nih.gov, accessed on 20 May 2024) using the Basic Local Alignment Search Tool program (BLAST, https://blast.ncbi.nlm.nih.gov/Blast.cgi, accessed on 20 May 2024). The phylogenetic tree was developed in MEGA11 based on the 16S rDNA sequence by the neighbor-joining method, and the bootstrap replications were 1000 in the conduction.

### 3.6. APP Degradation Conditions

For the study of phosphate resources, the initial concentrations of K_2_HPO_4_ in the R2A medium were set to 0 and 0.3 g/L. Two single-factor experiments were conducted to study the APP degradation characteristics of *Acinetobacter nosocomialis* D-3 by changing the culture temperature and initial pH of the R2A medium. For the temperature factor, different groups of *Acinetobacter nosocomialis* D-3 were incubated at 27 °C, 37 °C, and 47 °C environments in incubators. For the pH factor, the initial pH of the media was accommodated to 6, 7, and 8 using 2 M NaCl and 1 M NaOH. Orthogonal experiments with temperature and pH were performed. When the two factors were not subjects of the experiment, the initial JLS-PNA220-A concentration, pH, and culture temperature were set to 12 mg/L, 7.3, and 37 °C, respectively. In contrast, the other cultural factors remained consistent.

## 4. Conclusions

This study presents a pioneering approach for assessing APP concentration, leveraging the symbiotic interplay between DAPI and the PolyP structure. Specifically, the *N*-containing indole ring of DAPI intricately interacts with the O atoms of phosphate, culminating in the formation of DAPI/PolyP complexes. These complexes emit characteristic fluorescence with a discernible peak at 550 nm upon excitation at 330 nm, directly mirroring the concentration of APP. It showcases remarkable sensitivity and an impressively low LOD of 3.079 μg/L, rendering it a swift and precise method for APP detection. Moreover, its potential extends to monitoring the APP content in flame-retardant products, such as JLS-PNA220-A, thereby facilitating comprehensive evaluations of flame-retardant efficacy. Furthermore, the above assay provides an accurate and efficient method for assessing the biodegradation of APP flame retardants and establishes a feasible environmental remediation strategy. An adept APP-degrading strain, named D-3, was meticulously isolated from sewage sludge. Through meticulous morphological characterization and 16S rDNA sequencing, strain D-3 was taxonomically classified within the *Nosocomialis* genus. A detailed examination of strain D-3’s APP degradation prowess under diverse incubation conditions revealed optimal parameters: an incubation temperature of 37 °C and a pH of 7. Under these conducive conditions, a rapid degradation phase was observed over two days, culminating in approximately 85% degradation within four days. The exemplary proficiency displayed by *Acinetobacter nosocomialis* D-3 in APP degradation underscores its potential as an eco-friendly agent for remediating residual APP through environmentally benign biodegradation processes. Moreover, its utilization is promising for optimizing wastewater treatment in industrial settings, fostering sustainable environmental management practices.

## Data Availability

Data are contained within the article and Supplementary Materials.

## Supplementary Materials

The following supporting information can be downloaded at: https://www.mdpi.com/article/10.3390/molecules29112667/s1, Figure S1: The fluorescence intensity of DAPI was observed as the incubation time increased from 1 to 8 min in the presence of 3 mg/L APP and 0.5 μM DAPI. *Ex* = 330 nm, and *Em* = 550 nm. Figure S2: (a) Fluorescence emission intensity of DAPI as APP concentrations ranging from 0 to 3000 μg/L. The final APP concentrations were set as follows: 0, 30, 90, 150, 240, 300, 900, 1500, 2400, and 3000 μg/L. (b) The linear relationship between the relative fluorescence intensity (F-F0) in 550 nm and APP concentration spanning from 0 to 300 μg/L. The final concentration of DAPI was 0.5 μM. *Ex* = 330 nm; *Em* = 550 nm. Figure S3: Gram staining of strain D-3, observing under optical microscope (a) amplification ×100. (b) The area in the red frame of (a) is magnified ×3. Table S1: Orthogonal array experimental designed for APP degradation optimization of *Acinetobacter nosocomialis* strain D-3.
