# Supplementary material for: Monitoring Ammonium Polyphosphate (APP) Biodegradation by Acinetobacter nosocomialis D-3 Using DAPI
Source: Molecules. 2024 Jun 5;29(11):2667. doi: 10.3390/molecules29112667 (PMC11173948; doi:10.3390/molecules29112667)
Supplement: Supplementary file 1 [file molecules-29-02667-s001.zip › molecules-2994568-supplementary.pdf]

Monitoring Ammonium Polyphosphate (APP) Biodegradation by *Acinetobacter nosocomialis* D-3 Using DAPI

Xiangxiang Li [1,†], Yule Cai [1], Qiqing Qiu [2,†], Jiamin Wu [1], Jing Wang [2] and Jieqiong Qiu [1,*]

[1] College of Life Sciences and Medicine, Zhejiang Sci-Tech University, Hangzhou 310018, China
[2] Hangzhou JLS Flame Retardants Chemical Co., Ltd., Hangzhou 310011, China
* Correspondence: qiujieqiong@zstu.edu.cn; Tel.: +86-0571-86843192
† These authors contributed equally to the work.

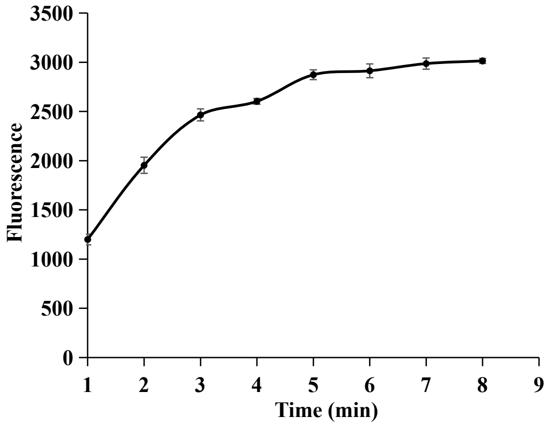

Figure S1. The fluorescence intensity of DAPI was observed as the incubation time increased from 1 to 8 min in the presence of 3 mg/L APP and 0.5 μM DAPI. *Ex* = 330 nm, and *Em* = 550 nm.

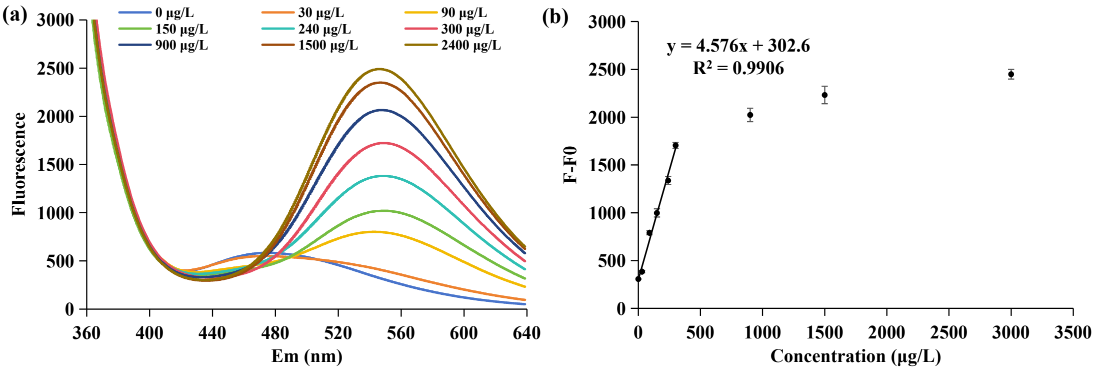

Figure S2. (a) Fluorescence emission intensity of DAPI as APP concentrations ranging from 0 to 3000 μg/L. The final APP concentrations were set as follows: 0, 30, 90, 150, 240, 300, 900, 1500, 2400, and 3000 μg/L. (b) The linear relationship between the relative fluorescence intensity (F-F0) in 550 nm and APP concentration spanning from 0 to 300 μg/L. The final concentration of DAPI was 0.5 μM. *Ex* = 330 nm; *Em* = 550 nm.

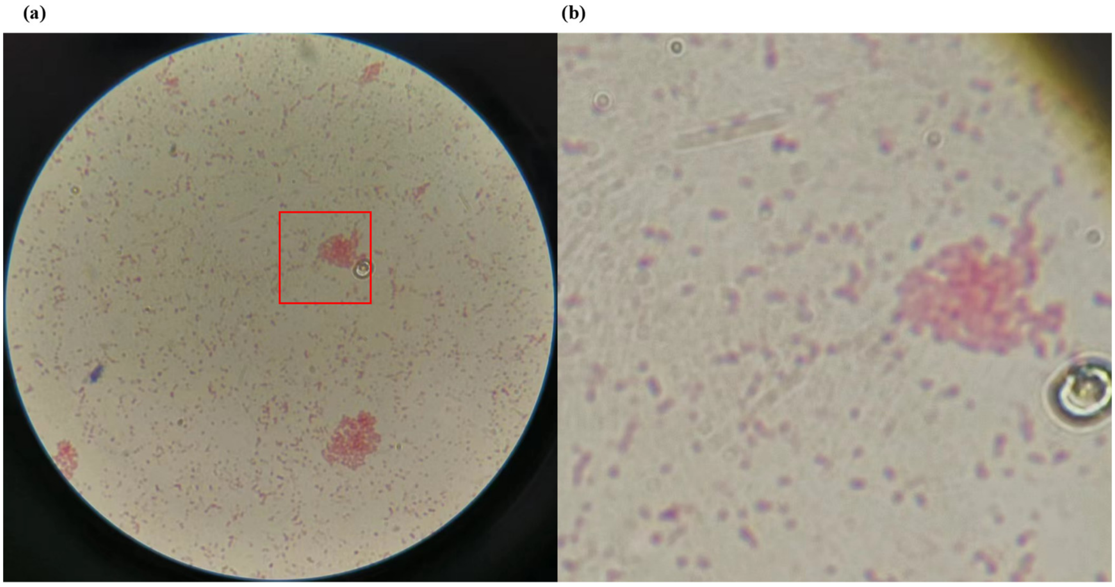

Figure S3. Gram staining of strain D-3, observing under optical microscope (a) amplification ×100. (b) The area in the red frame of (a) is magnified ×3.

Table S1. Orthogonal array experimental designed for APP degradation optimization of *Acinetobacter nosocomialis* strain D-3.

Test	Temperature (°C)	pH	Fluorescence intensity (F-F0)	Degradation rate (%)
1	27	6	647.8	76.10
2	27	7	650.7	75.73
3	27	8	660.8	74.42
4	37	6	593.9	83.08
5	37	7	574.3	85.62
6	37	8	577.3	85.23
7	47	6	895.7	44.01
8	47	7	791.3	57.52
9	47	8	809.9	55.12
